# Evidence for relaxed selection of mitogenome in rapid-flow cyprinids

**DOI:** 10.1007/s13258-019-00817-7

**Published:** 2019-04-23

**Authors:** Yao Lu, Hu Xing, Dongsheng Zhang

**Affiliations:** 1 National Demonstration Center for Experimental Fisheries Science Education, Shanghai Ocean University, Shanghai, 201306 People’s Republic of China; 2 Shanghai Collaborative Innovation for Aquatic Animal Genetics and Breeding, Shanghai Ocean University, Shanghai, 201306 People’s Republic of China; 3 Key Laboratory of Freshwater Aquatic Genetic Resources, Ministry of Agriculture, Shanghai Ocean University, Shanghai, 201306 People’s Republic of China

**Keywords:** Relaxed selection, Cyprinid, Mitogenome, Rapid-flow

## Abstract

**Background:**

Hypoxia adaptation is developed in many fish species, which helped them to habitat most of water bodies. However, fishes living under high oxygen concentration may lose this feature. Rapid flows provide high level and stable dissolved oxygen, which facilitate organism’s oxygen supply and energy production. Previous studies showed that fish species from rapid-flow habitats exhibited lower hypoxia tolerance compared with fish from intermediate- and slow-flow habitats. Mitochondrial genomes code 13 key components in oxidative phosphorylation pathway; these genes may be under relaxed selection in rapid-flow species.

**Objectives:**

The primary objectives of this study is to investigate the evolutionary patterns of the 13 mitochondrial OXPHOS genes among nine cyprinids from different water bodies and to test the hypotheses that mitochondrial OXPHOS genes may experience relaxed selection in rapid-flow habitats.

**Methods:**

We classified nine cyprinid fish species into three groups based on their habitats: rapid-flow, intermediate-flow and slow-flow. To detect relaxed selections, we investigated the 13 protein-coding genes with codon evolution programs RELAX; to estimate evolutionary rates among the cyprinids, free-ratio model in Codeml program was applied; Branch-site models were applied to detect positive selection sites. The polymorphisms of homologous sites were evaluated with PROVEAN program and projected to 3D structure prediction of the proteins using SWISS-MODEL.

**Results:**

We found that nine out of the 13 genes are under relaxed selection in rapid-flow species. Furthermore, dN, dS and dN/dS are relatively increased when compared with those of intermediate-flow species. More amino acid polymorphic sites are presented in rapid-flow species than in intermediate- and slow-flow species. Furthermore, rapid-flow species had more deleterious substitutions than other groups. 3D structure prediction of these proteins and projection of the polymorphic sites indicated that these sites were randomly distributed, suggesting relaxed functional constraints of these proteins in rapid-flow species.

**Conclusion:**

Our results suggest that mitochondrial genes are under relaxed selection in rapid-flow cyprinids.

## Introduction

Adaptation to ambient environment is the key for organisms to survive and thrive, but the relationship between phenotypic adaptation and molecular evolution remains poorly understood. As one of the major environmental constraints, hypoxia occurs naturally in aquatic systems due to all kinds of natural and anthropogenic causes, including diurnal oscillations in algal respiration and eutrophication. Fish habitat in most water bodies on earth has great variation in hypoxia tolerance. Hypoxia-tolerant fish species developed many unique adaptive strategies. Some may reduce oxygen consumption by shifting metabolic pathways and regulating expression of key genes, on the other hand, some will increase oxygen delivery by stimulating angiogenesis and regulating the proliferation of red blood cells (Nikinmaa 2005). Recent transcriptomic studies on fish adaptation to hypoxia provided us insights into molecular evolution and adaption to hypoxia (Yang et al. 2014a,b; Zhang et al. 2017). Species living under high oxygen concentration may partially lose the ability in hypoxia adaptation, but the underlying molecular mechanisms are not well known (Fu et al. 2014).

As the ultimate oxygen consumer, mitochondria consume about 95% of oxygen in aerobic respiration through oxidative phosphorylation (OXPHOS). Thus, it is critical for hypoxia-tolerant species to improve their efficiency of oxygen usage under hypoxia. The mitochondrial genome encodes 13 essential OXPHOS system proteins (seven subunits of the NADH dehydrogenase complex, three subunits of the cytochrome c oxidase, two subunits of ATP synthase, and the cytochrome b subunit of the cytochrome bc1 complex). It has been well established that positive and negative selection acts on these proteins (Shen et al. 2010; Menezes et al. 2013). As oxygen consumption and energy production are critical for “high performance” species, OXPHOS subunits are thought to be under strict functional constraints in these species (Dalziel et al. 2006; Sun et al. 2011). On the other hand, a relaxed selection of energy metabolism genes has been suggested for sedentary species as energy production are less critical for them (Shen et al. 2009).

This paper aims to investigate the evolutionary patterns of the 13 mitochondrial OXPHOS genes among nine cyprinids from different water bodies. Cyprinid fishes are the largest group of fresh-water fishes with wide geographic distribution. A previous study on cyprinid fishes from rapid-, slow- and intermediate-flow habitats showed that hypoxia tolerance is related to habitat (Fu et al. 2014). As rapid flows generally exhibit stable and high level dissolved oxygen (DO) concentration, whereas intermediate- and slow-flows exhibit low DO levels and large DO fluctuation. They found that rapid-flow habitats have a limited capability of hypoxia adaptation when compared with fish from intermediate- and slow-flow habitats (Fu et al. 2014). Since Dissolved Oxygen concentration is directly related to oxygen consumption and energy production, we hypotheses that mitochondrial OXPHOS genes may experience relaxed selection in rapid-flow habitats.

## Materials and methods

### Data collection and sequence alignment

**Fig. 1:**
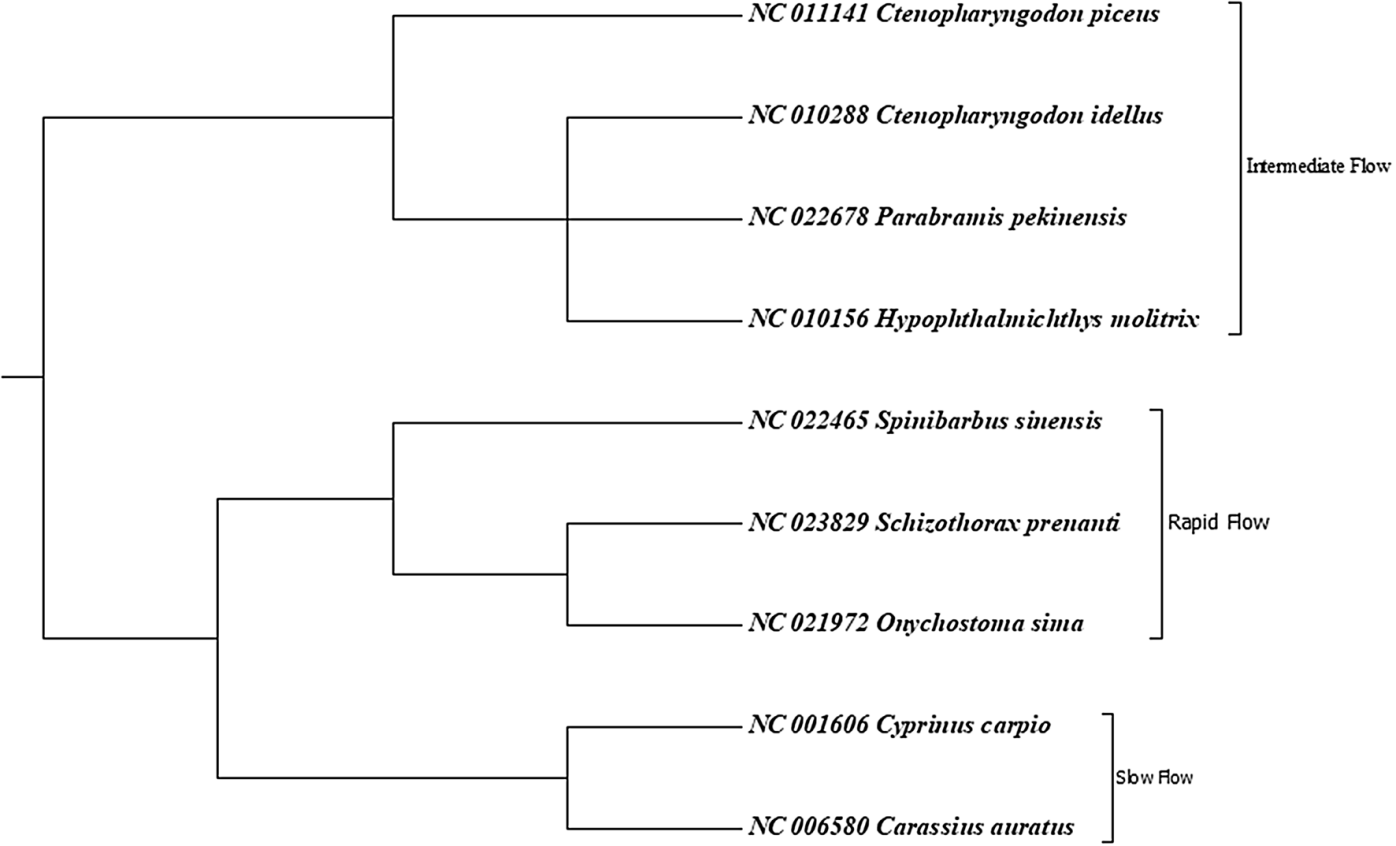
Phylogenetic relationships among the species investigated in this study

Nine closely related cyprinid species (*Schizothorax prenanti*, *Onychostoma sima*, *Spinibarbus sinensis*, *Carassius auratus*, *Cyprinus carpio*, *Hypophthalmichthys molitrix*, *Parabramis pekinensis*, *Ctenopharyngodon idellus*, and *Ctenopharyngodon piceus*) were classified into three groups (Fu et al. 2014): rapid-flow species, intermediate-flow species and slow-flow species (Fig. 1). Their mitogenome sequences were downloaded from NCBI database with accession numbers shown in Fig. 1. The coding DNA sequences for the 13 genes were aligned using MUSCLE (Edgar 2004) codons alignment with MEGA 7 (Kumar et al. 2016) and corrected manually according to constraints imposed by the sequence conservation among the species.

### RELAX analysis

To test for evidence of relaxed selection in the 13 genes, we used the program RELAX, which is designed for detecting relaxed selection between two groups in a codon-based phylogenetic framework (Wertheim et al. 2014). The sequence alignments were uploaded to RELAX online server (https://www.datamonkey.org/RELAX). The vertebrate mitochondrial translation code was applied for the RELAX analysis. Branches of rapid-flow, intermediate-flow and slow-flow were respectively assigned as test branches to investigate relaxed/intensification selection for these branches.

### Selection analysis

To characterize selection constraints on the 13 genes for different habitats, The CODEML program from PAML4.8 (Yang 2007) with the free-ratio model (model = 1) was run on each gene and a concatenation of all alignments of the 13 genes. The guide tree used in the analysis was modified according to a previous study (Wang et al. 2007) with MEGA7 (Kumar et al. 2016) (Fig. 1).Parameters, including dN, dS, dN/dS, were obtained for each terminal node. Furthermore, branch models in CODEML were applied to investigate the evolutionary rates of lineages of rapid-flow, intermediate-flow and slow-flow species, the fishes of the three groups were respectively assigned as foreground and compared with null model, which assumes that all branches have the same evolutionary rate. Likelihood ratio tests (LRTs) were applied to test if there were significant evolutionary rate differences between foreground and background lineages.

To explore variation across codons of each gene, we compared the likelihood of fit of evolutionary models implemented in CODEML site models and tested positive selection using model comparison between models allowing and not allowing positive selections, namely the M1a–M2a, M7–M8 and M0–M3 comparisons. To search for positive selection at individual sites along specific lineages, we used two variants of the Branch-site model A and the LRT between them (Model A and Model A modified) (Zhang et al. 2005). Each of the three groups was taken as foreground and compared to the other two groups (background) in branch-site analysis, respectively. The P-values were computed based on the Chi-square statistic and genes with P-value less than 0.05 were treated as candidates for positive selection.

### Amino acid sequence alignment analysis and PROVEAN analysis

The Protein Variation Effect Analyzer (PROVEAN) (Choi et al. 2012) was employed to assess the functional effect of every fixed amino acid change. The confidence threshold of − 2.5 was used to determine if an amino acid replacement is likely to have an effect on protein function. The reconstructed ancestral sequences of the 13 genes were used as a template, respectively, and every fixed amino acid replacement present in each species was used as a query.

### Three-dimensional structure prediction

Three-dimensional models of the 12 homologs (except ATP8) were constructed with Swiss-Model software (Biasini et al. 2014) and visualized using PYMOL.

### Statistical analysis

Significant differences between groups of data were determined using t-tests implemented in MS EXCEL and R.

## Results

### RELAX analysis

The most interesting results came from RELAX analysis. When rapid-flow species were taken as test branches, nine genes were identified under relaxation, including three subunits of the cytochrome c oxidase and six subunits of the NADH dehydrogenase complex (Table 1). Five and three genes were identified as intensification when intermediate-flow and slow-flow species were taken as test branches, respectively (Table 1).

**Table 1:** Genes identified under relaxation and intensification when different branches are tested with RELAX

Selection	Test branches	Gene	P value
Relaxation	Rapid-flow	COX1	0.03
		COX2	0.00004
		COX3	0.0003
		ND1	0.0004
		ND2	0.03
		ND3	0.02
		ND4l	0.0007
		ND5	0.00004
		ND6	0.02
Intensification	Intermediate-flow	COX2	0.0007
		COX3	0.01
		ND1	0.002
		ND4l	0.01
		ND5	0.0003
	Slow-flow	COX2	0.04
		COX3	0.008
		ND6	0.008

### Variation in dN, dS and dN/dS

**Fig. 2:**
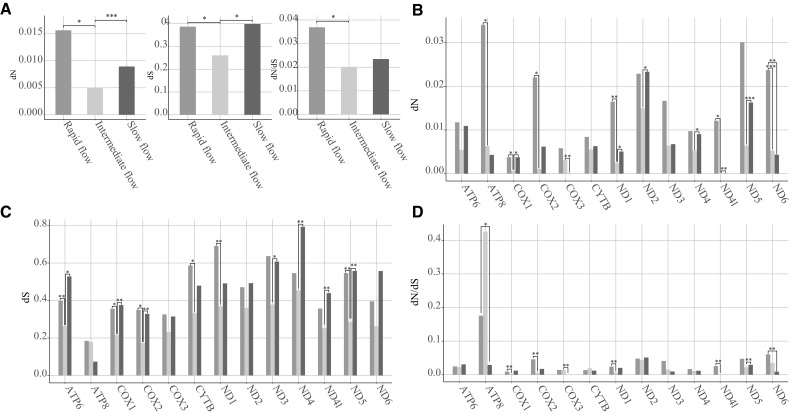
Comparisons of dN, dS and dN/dS values. **a** dN, dS and dN/dS values for concatenated alignment of the 13 genes. **b**, **c**, **d** dN, dS and dN/dS values for individual homologous genes, respectively. Blue boxes depict the values for rapid-flow fish, orange boxes for intermediate-flow fish and green boxes for slow-flow fish. *:0.10 > P > 0.05; **:0.05 > P > 0.01; ***: P < 0.01 (colour figure online)

Selection constraints on different lineages were tested using free-ratio model in CODEML program. We averaged dN, dS and dN/dS values for the concatenated alignment of the 13 genes, and compared these parameters of the three groups using t-tests. Our data indicated that non-synonymous substitution rate is relatively high in rapid-flow species but relatively low in intermediate-flow species. As shown in Fig. 2a, dN and dS of intermediate-flow species were marginally significantly lower (P-values were between 0.05–0.10) or significantly lower than the corresponding values of rapid-flow and slow-flow species; and dN/dS of intermediate-flow species was also marginally significantly lower than that of slow-flow species.

Investigation on individual homologous genes also showed similar trends. Six out of the 13 genes showed significantly or marginally significantly higher dN values in rapid flow species than those for intermediate-flow species (Fig. 2b). Six and four genes have higher dS and dN/dS values in rapid-flow species than those in intermediate-flow species, respectively (Fig. 2c, d).

We investigated the evolutionary rates of the three groups respectively by comparing with the null hypothesis that assumes the same evolutionary rate for each lineages. We found that rapid-flow species showed significantly increased evolutionary rate than other lineages (Pairwise t-test, P < 0.05), while intermediate-flow species showed s significantly decreased evolutionary rate than other lineages (Pairwise t-test, P < 0.05). These results is consistent with the results drawn from free-ratio models. To explore positive selection sites in the 13 genes, we applied site model and branch-site model. No positive selection sites were identified in the analyses (data not shown).

### Amino acid substitution pattern

**Fig. 3:**
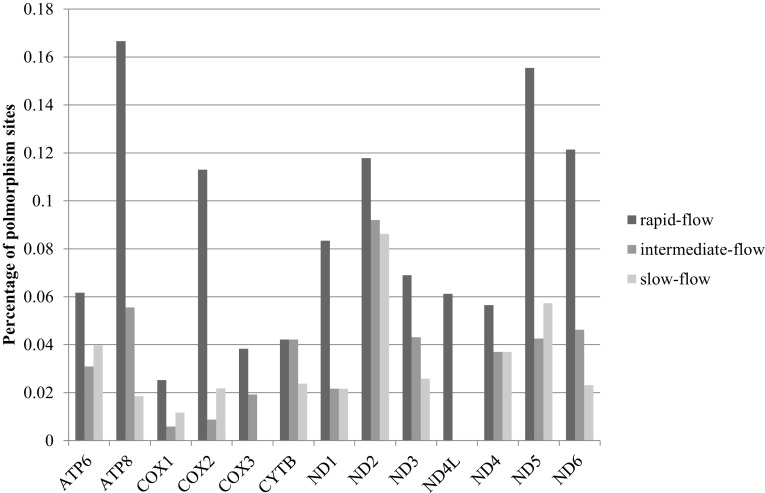
Frequencies of Amino acid polymorphism sites in different groups

We further investigated amino acid polymorphisms among the three groups. We noticed that some sites are conserved in both slow- and intermediate-flow species but diverse in the rapid-flow species. For example, each species from rapid-flow group has a unique amino acid (Cys, Ser and Pro) at the site 447 for ND5 but only one conserved amino acid (Ser) present at the same site in both slow- and intermediate- flow species. The rapid-flow species have more polymorphism sites than the other two groups, as shown in Fig. 3. Among the 3798 homologous sites of the 13 genes, there are significantly more polymorphic sites (312) present in rapid-flow species than in intermediate- (131) and slow-flow (126) groups (pairwise t-tests, P-values are 0.015 and 0.004, respectively).

**Fig. 4:**
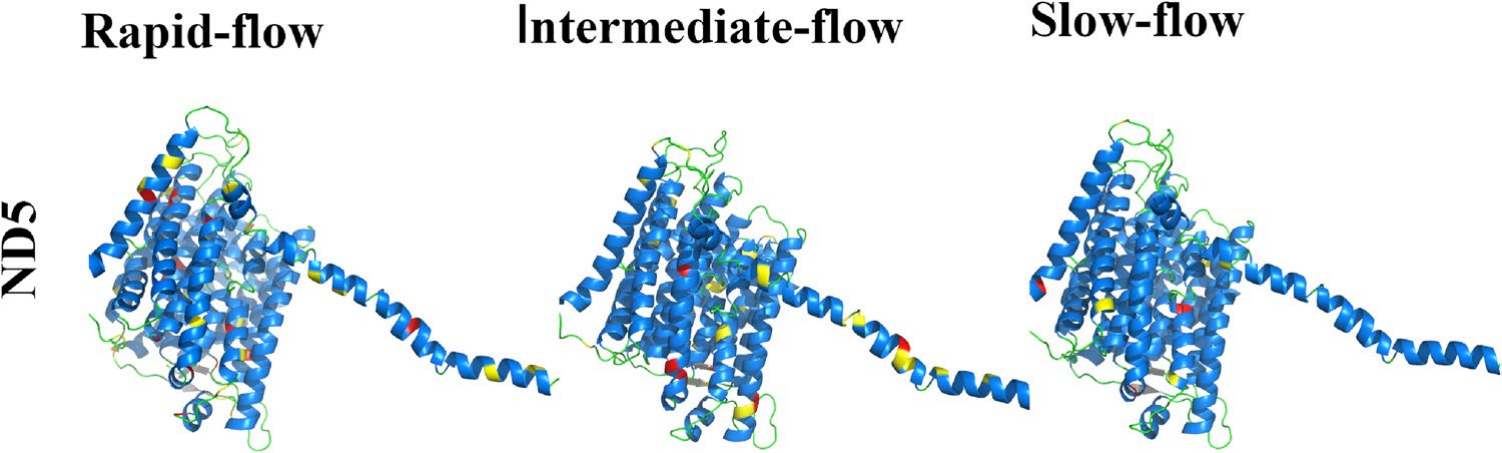
3D structure prediction of ND5 and projection of polymorphism sites in 3D model. Yellow: neutral substitution sites. Red: deleterious substitution sites (colour figure online)

The potential effects of amino acid polymorphisms on protein structure and function were explored with PROVEAN software. We detected 60, 19 and 32 deleterious substitutions present in rapid, intermediate- and slow-flow species. The 3D structures of 12 proteins were predicted (3D structure of ATP8 was not predicted due to its short length), and the polymorphic sites were projected in the 3D models of these proteins. As shown in Fig. 4, the polymorphic sites of ND5 are presented in all secondary structure elements.

## Discussion

Relaxed selection is a key issue for speciation and evolution but not well understood. It occurs when environmental change eliminates or weakens a challenging demand which was formerly critical for the maintenance of a particular biological feature (Lahti et al. 2009). In terms of fish, oxygen supply is a critical environmental factor for survival due to limited dissolved oxygen in water. Comparing with other water bodies, Rapid-flow provides relatively high concentration and stable dissolved oxygen, which may lead to relaxed selection of respiration-related genes in the rapid-flow species.

In this study, we reported evidence for relaxation of purifying selection in the coding genes of the mitogenome in rapid-flow cyprinids. The first piece of significant evidence was found with RELAX analysis. Nine out of 13 genes showed significant relaxed selection in rapid-flow species when comparing with intermediate- and slow-flow species. Furthermore, our results indicated increases of dN and dS in rapid-flow species, which suggested accelerated evolution for both non-synonymous and synonymous substitution are presented in these species. Meanwhile, intermediate-flow species showed decreased rates for dN, dS and dN/dS when comparing with the other two groups, which may be due to intensified purifying selection on the mitochondrial genes in these species. Accelerated evolutionary rate is related to relaxed purifying selection of genes (Ometto et al. 2012; Strohm et al. 2015), our results clearly showed that genes encoded by mitogenomes tend to be under relaxed selection in rapid-flow cyprinids. Interestingly, a study on flightless insects indicated that flight loss is related to relaxed selection of the 13 genes encoded by mitogenomes (Mitterboeck et al. 2017). Meanwhile, some studies showed that mitochondrial DNA protein-encoding genes are subject to positive selection in Flying Insects (Li et al. 2018; Yang et al. 2014a,b). These studies, together with our results, indicate that evolution of mitochondrial energy metabolism genes play important roles in adaptation of organisms.

Another piece of evidence came from amino acid polymorphisms in the 13 genes. We detected more amino acid changes in rapid-flow species than intermediate- and slow-flow species. Furthermore, more deleterious changes were found in rapid-flow species than the other two groups, which indicated that functional constraints of these genes are relaxed in rapid-flow species. Contrary to adaptive changes, which enhance the functionality of protein under environmental stress (Fields et al. 2015), deleterious changes in protein sequences indicated that these rapid-flow species face a less stressful environmental changes, i.e., adequate oxygen supply for these species.

Fu et al. (2014) found that fish species from rapid-flow habitats were more vulnerable to acute hypoxia exposure, but the molecular mechanism has not been explored. One possible reason may be that the decreased functionality of the respiration chain in these species lead to low hypoxia tolerance. Our results indicate that mitochondrial energy metabolism genes are under relaxed purifying selection in rapid-flow species, which may explain the decreased functionality of the respiration chain. Our results thus bear both on studies of hypoxia adaptation and on studies of gene family evolution in general. We have to point out that we do not have a large number of species to draw a more solid conclusion. Furthermore, molecular biology studies on these proteins may reveal more functional effects of the substitutions occurred in rapid-flow species. Despite its preliminary character, this study clearly indicates that relaxed selection act on the mitogenome-coding genes in rapid-flow fishes. Further studies on more energy metabolism related genes and more species will give us more solid evidence on relaxed selection of genes involved in oxygene-comsuming biological processes in rapid-flow fishes.
